# The Origin of Catalytic Benzylic C−H Oxidation over a Redox‐Active Metal–Organic Framework

**DOI:** 10.1002/anie.202102313

**Published:** 2021-06-04

**Authors:** Louis Kimberley, Alena M. Sheveleva, Jiangnan Li, Joseph H. Carter, Xinchen Kang, Gemma L. Smith, Xue Han, Sarah J. Day, Chiu C. Tang, Floriana Tuna, Eric J. L. McInnes, Sihai Yang, Martin Schröder

**Affiliations:** ^1^ Department of Chemistry University of Manchester Manchester M13 9PL UK; ^2^ Photon Science Institute University of Manchester Manchester M13 9PL UK; ^3^ Diamond Light Source Harwell Science Campus Oxfordshire OX11 0DE UK

**Keywords:** benzylic oxidation, catalysis, copper, electron paramagnetic resonance, metal–organic framework

## Abstract

Selective oxidation of benzylic C−H compounds to ketones is important for the production of a wide range of fine chemicals, and is often achieved using toxic or precious metal catalysts. Herein, we report the efficient oxidation of benzylic C−H groups in a broad range of substrates under mild conditions over a robust metal–organic framework material, MFM‐170, incorporating redox‐active [Cu_2_^II^(O_2_CR)_4_] paddlewheel nodes. A comprehensive investigation employing electron paramagnetic resonance (EPR) spectroscopy and synchrotron X‐ray diffraction has identified the critical role of the paddlewheel moiety in activating the oxidant ^t^BuOOH (tert‐butyl hydroperoxide) via partial reduction to [Cu^II^Cu^I^(O_2_CR)_4_] species.

## Graphical abstract

The oxidation of benzylic C−H groups in a broad range of substrates under mild conditions has been achieved using a robust metal–organic framework, MFM‐170, incorporating redox‐active [Cu_2_^II^(O_2_CR)_4_] paddlewheel nodes. EPR spectroscopy and synchrotron X‐ray diffraction have identified the critical role of the paddlewheel moiety in activating the oxidant ^*t*^BuOOH (*tert*‐butyl hydroperoxide) via partial reduction to [Cu^II^Cu^I^(O_2_CR)_4_] species.

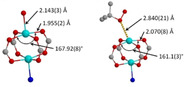

The selective oxidation of benzylic compounds is widely employed in chemical industry for the production of ketones and numerous fine chemicals.^[1]^ Conventional homogeneous processes use stoichiometric amounts of transition metal complexes [e.g., Cr^VI^ and Mn^VII^], which have significant environmental implications and result in costly procedures for waste treatment.^[2]^ In contrast, heterogeneous catalysts typically consisting of precious metal nanoparticles supported on porous materials (e.g., zeolites, silica, titania) have been used extensively for a wide variety of large scale catalytic reactions.^[3]^ However, the nature of the dispersion within these supported materials makes them prone to deactivation via aggregation and leaching of the active metal.^[4]^ Therefore, the development of new efficient heterogeneous catalysts based upon porous materials with immobilised, uniformally‐ and atomically‐dispersed active metal sites remains an important and challenging target.

Metal–organic framework (MOF) materials consist of single‐site metal nodes bridged by organic linkers, and can effectively hinder the aggregation and leaching of metal sites. Thus, they have shown great promise in catalysis.^[5]^ In recent years, MOFs and their composites have proven to be active catalysts in various organic transformations including hydrogenations,[^6^, ^7^] Knoevenagel condensations,[^8^, ^9^, ^10^] cross coupling reactions,[^7^, ^11^] cyanosilylations,^[12]^ Friedel–Crafts reactions^[12]^ and oxidations.[^7^, ^13^, ^14^, ^15^, ^16^] However, the determination of reaction intermediates in these MOF‐catalysed reactions is a major challenge not least because binding of substrates often undergoes complex, dynamic processes with short‐lived intermediates and radicals. Here, we report a study on the use of MFM‐170, constructed from redox‐active [Cu_2_^II^(O_2_CR)_4_] paddlewheels linked by a pyridyl functionalised tetracarboxylate ligand,^[17]^ in combination with an external oxidant, as a highly active catalyst for oxidation of benzylic C−H functions. The redox behaviour of [Cu_2_^II^(O_2_CR)_4_] paddlewheels and the detection of intermediate radicals have been investigated by EPR spectroscopy. The selective activation of the oxidant ^*t*^BuOOH (*tert*‐butylhydroperoxide) on the paddlewheel has been interrogated by synchrotron X‐ray powder diffraction (SPXRD).

MFM‐170, [Cu_2_(C_33_H_17_NO_8_)(H_2_O)]⋅3 DMF, was synthesised via our previously reported method.^[17]^ SEM images indicate a Bilinski dodecahedron morphology with a wide crystal size distribution between 1 and 65 μm (Figure S1). MFM‐170 forms a self‐interpenetrated (3,36)‐connected network with [Cu_2_(O_2_CR)_4_] paddlewheels connected through carboxylate groups bound in the equatorial plane. The axial positions of the paddlewheel are occupied by a pyridyl N‐donor on one Cu^II^ and a water molecule on the other. The bound H_2_O can be removed by heating under vacuum to generate a coordinatively unsaturated Cu^II^ site.^[17]^ The phase purity of the bulk material was confirmed by powder X‐ray diffraction (PXRD) and TGA (Figures S2 and S3). The fully desolvated MOF shows an apparent surface area of 2325 m^2^ g^−1^ as determined from the N_2_ sorption isotherm (Figure S4).

The oxidation of indane was studied to assess the catalytic activity of MFM‐170 towards the oxidation of the benzylic C−H group. While negligible oxidation of indane was observed using H_2_O_2_ or O_2_ alone, MFM‐170 displayed remarkable activity in the presence of ^*t*^BuOOH. A series of tests were conducted to optimise the reaction conditions: a yield of 94 % of 1‐indanone was achieved by reaction of indane with MFM‐170 (10 mol %), ^*t*^BuOOH (3 equiv) at 65 °C for 1 day (Table 1, entry 5). In the absence of ^*t*^BuOOH, negligible conversion of indane was observed (Table 1, entry 1), and without MFM‐170 the oxidant is thermally activated and offers a moderate conversion of indane to a mixture of the alcohol and ketone (Table 1, entry 2), demonstrating the critical role of MFM‐170 in activating ^*t*^BuOOH in a selective manner. The time–conversion plot confirms that there is no induction period and a rapid conversion of indane is observed within the first 5 h with a diminished instantaneous rate as the reaction proceeds (Figure 1 a). The activity of homogeneous catalysts based upon simple Cu^II^‐salts including CuCl_2_, Cu(NO_3_)_2_ and Cu(OAc)_2_ was also studied under optimised conditions, and afforded 1‐indanone in yields of 34, 58 and 63 %, respectively, lower than that (94 %) over MFM‐170 (Figure S5). This result demonstrates that immobilising the Cu^II^ ions into the paddlewheel moiety of the MOF drastically enhances the catalytic stability and activity towards benzylic oxidations by preventing their aggregation and deactivation. The reusability of MFM‐170 was studied (Figure S6) over 5 cycles to give yields of 1‐indanone in the range 87–94 %. The variation in intensities of some Bragg peaks of recycled catalysts is likely due to the inclusion of guest molecules in the pore^[17]^ and/or some minor local structural change after reaction (Figure S7); the latter is consistent with a small reduction of the apparent surface area (2076 m^2^ g^−1^; Figure S4). Elemental analytical data suggest the leaching of Cu sites into solution is within the detection limit of 0.5 % (Table S1). Removal of the catalyst by filtration at approximately 30 % conversion led to a further production of 1‐indanone of about 20 %, which is likely owing to the thermal activation of the oxidant consistent with the blank experiments (Figures 1 b, S8).

**Figure 1:**
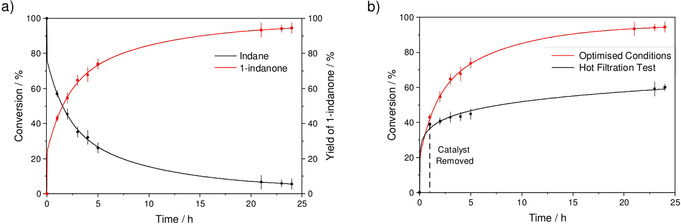
a) Plot of time vs. conversion for the oxidation of indane catalysed by MFM‐170 showing the conversion of indane (black) and the percentage of 1‐indanone formed (red) over time. b) Reaction profiles for the optimised conditions (red) and hot filtration, leach test (black). Reaction conditions: indane (0.25 mmol), MFM‐170 (0.025 mmol, 0.05 mmol Cu), ^*t*^BuOOH (0.75 mmol), MeCN (4 mL) 65 °C, 24 h.

**Table 1:** Yields of indane oxidation catalysed by MFM‐170.^[a]^ 
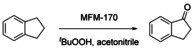

Entry	Catalyst loading [mol %]	^*t*^BuOOH (equiv)	*T* [°C]	Yield [%]	TOF^[b]^
1	10	–	65	2	8
2	–	3	65	31	n/a
3	1	3	65	82	3416
4	5	3	65	87	725
5	10	3	65	94	392
6	20	3	65	92	192
7	10	3	50	87	363
8	10	3	60	93	388
9	10	3	70	90	375
10	10	3	80	94	392
11	10	1.5	65	53	221
12	10	6	65	98	408

[a] Reaction conditions unless noted otherwise: indane (0.25 mmol), MFM‐170 (0.025 mmol), ^*t*^BuOOH (0.75 mmol), MeCN (4 mL) 65 °C, 24 h. [b] TOF=1000×(mol_product_ mol_active Cu_^−1^ h^−1^).

The likely mechanism of catalysis involves the generation of ^*t*^BuO^.^ and ^*t*^BuOO^.^ radicals by reactions of Cu centres within the [Cu_2_(O_2_CR)_4_] paddlewheel.[^1^, ^18^, ^19^, ^20^] The reactive *tert*‐butoxy and *tert*‐peroxy derived radicals abstract a hydrogen atom from the benzylic position of the substrate, and the resultant C radical is then converted to the product via O‐transfer. Rietveld refinement of the SPXRD data of ^*t*^BuOOH‐loaded MFM‐170 confirmed binding of ^*t*^BuOOH to the vacant metal site within the [Cu_2_(O_2_CR)_4_] paddlewheel (Figure 2, Figure S9) with Cu⋅⋅⋅O of 2.840(2) Å. The geometry of one of the Cu centres in the paddlewheel is clearly distorted with the O‐Cu‐O bond angles decreasing from 167.9(8)° to 161.1(3)°. This is accompanied by an increase in the Cu−O(carboxylate) bond length from 1.955(2) to 2.070(8) Å. Bond valence sum calculations suggest an oxidation state change at this Cu center from 1.86 to 0.99 (Table S2), suggesting the formation of a mixed Cu^II^Cu^I^ [Cu_2_(O_2_CR)_4_] paddlewheel species (Figure 2). This is consistent with the proposed formation of Cu^I^ centers via activation of peroxide reagents by Cu^II^.[^18^, ^19^, ^20^] A significant increase in the concentration of ^*t*^BuOH was also detected by GC–MS in the reaction solution after 24 h, consistent with the proposed mechanism (Scheme 1).

**Figure 2:**
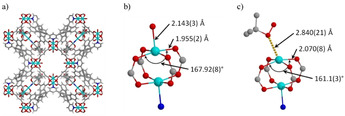
Views of a) the crystal structure of MFM‐170; b) the [Cu_2_(O_2_CR)_4_] paddlewheel in MFM‐170 with the pyridyl‐N donor shown in blue; c) ^*t*^BuOOH‐loaded MFM‐170.

**Scheme 1:**
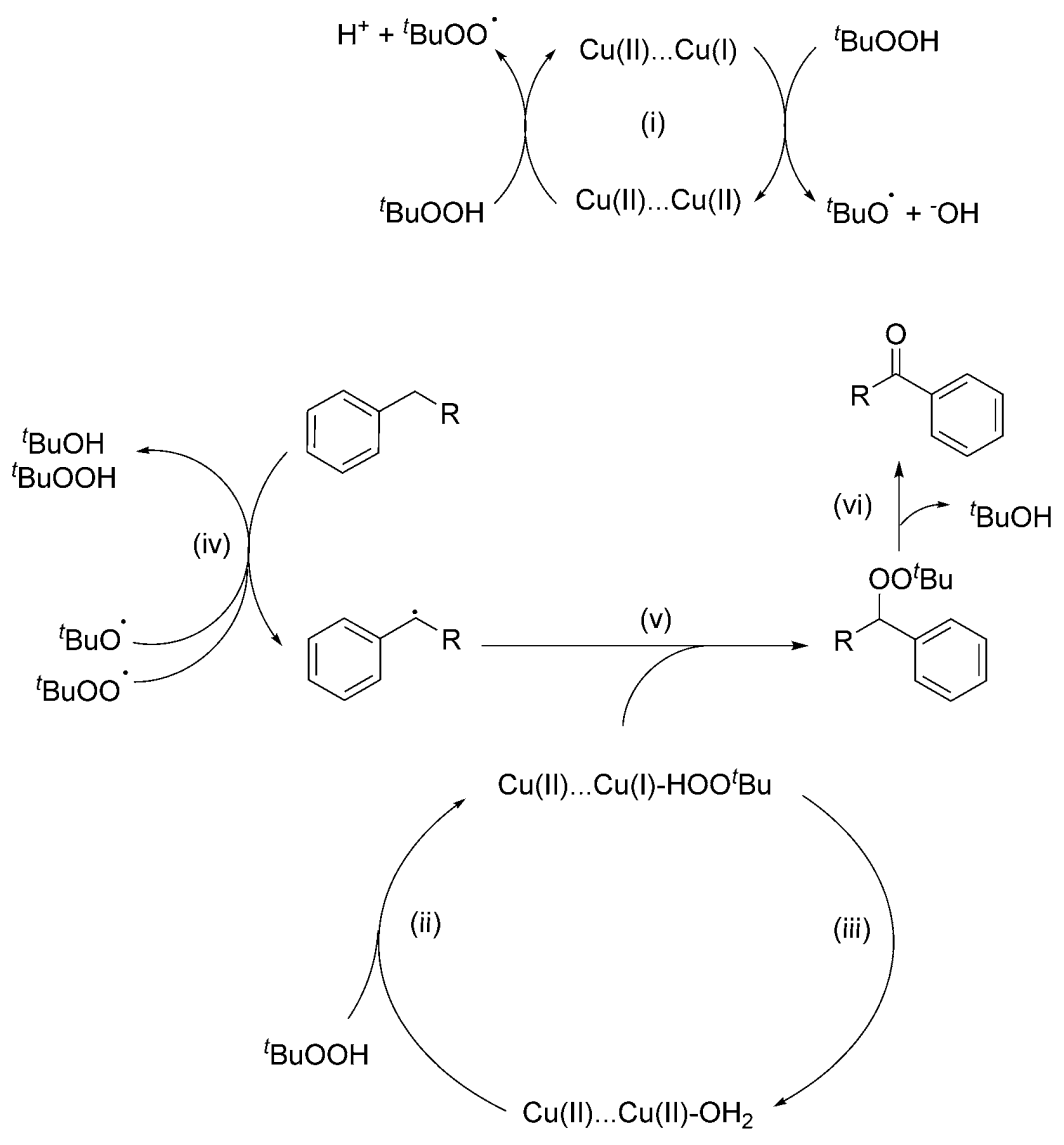
Proposed mechanism for the catalytic cycle.

The use of spin traps, such as *N*‐*tert*‐butyl‐α‐phenylnitrone (PBN),^[21]^ facilitates detection by EPR spectroscopy of short‐lived radicals that form during the catalytic process but which would otherwise be undetectable. Reaction of MFM‐170 and ^*t*^BuOOH in the presence of PBN in MeCN at 65 °C for 2 min gave an intense EPR signal at *g*=2.006, characteristic of PBN‐spin adducts. Thermally activated ^*t*^BuOOH gives only a very weak signal (Figures S10 and S11). This confirms that MFM‐170 activates ^*t*^BuOOH to generate intermediate radicals. In situ EPR measurements at 65 °C in the presence of PBN yielded narrower spectra that enabled unambiguous identification of both ^*t*^BuO^.^ and ^*t*^BuOO^.^ radicals (Figure 3 a, Table S3).[^22^, ^23^]

**Figure 3:**
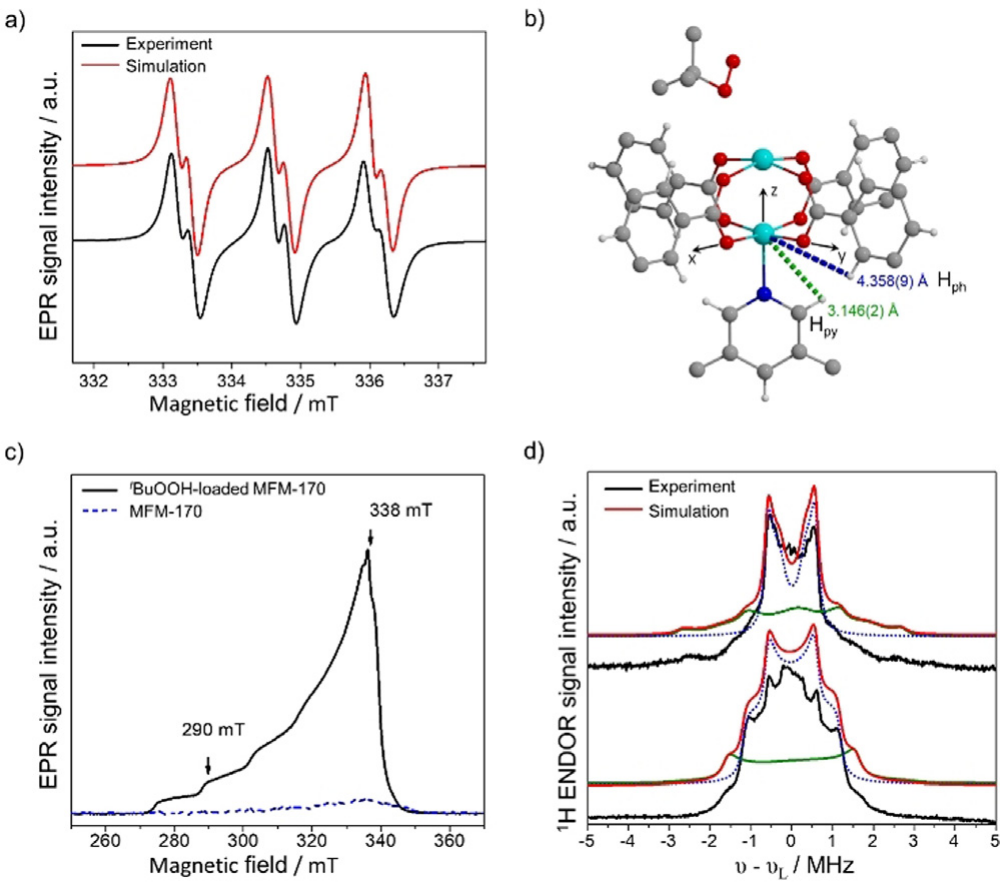
Views of a) X‐band (9.4 GHz) EPR spectrum of PBN spin trap with ^*t*^BuOOH and MFM‐170 in MeCN at 338 K (black), and simulation (red) of the combined spectrum of PBN‐^*t*^BuO^.^ radical (a_H_=2.25 G; a_N_=14.25 G) and PBN‐^*t*^BuOO^.^ radical (a_H_=1.4 G; a_N_=13.47 G; Figure S13 and Table S3 for details); b) structure of MFM‐170‐^*t*^BuOOH from SPXRD refinement, with definition of *x*,*y*,*z* molecular axes and crystallographic Cu⋅⋅⋅(Ar)H distances; c) X‐band electrically detected (ED) EPR spectra (9.724 GHz) at 5 K of MFM‐170 (blue) and ^*t*^BuOOH‐loaded MFM‐170 (black); d) X‐band (9.724 GHz) ^1^H ENDOR spectra of ^*t*^BuOOH‐loaded MFM‐170 at 290 and 338 mT (black) and their simulation using the point dipole model. Simulated ENDOR spectra (red) were obtained by summing simulations for the two ‐CH protons of the pyridine ring (H_py_; green) and the four ‐CH protons of the phenyl rings (H_ph_; blue; see text for details).

Binuclear Cu^II^ paddlewheel [Cu_2_(O_2_CR)_4_] moieties are generally EPR silent at low temperatures due to strong antiferromagnetic exchange between Cu^II^ centers (3d^9^, *s*=1/2 ) ions. This leads to a non‐magnetic *S=*0 spin ground state.[^24^, ^25^] However, if *one* of the Cu ions is reduced to 3d^10^ Cu^I^, as suggested by the structural analysis above, the EPR signal due to the remaining, uncoupled Cu^II^ *S*=1/2 ion will be observable. Continuous‐wave (CW) and pulse echo‐detected (ED) EPR spectra at 5 K of MFM‐170 give only very weak signals due to adventitious Cu^II^ impurities that are ubiquitous in polynuclear Cu^II^ species. On addition of ^*t*^BuOOH an intense EPR spectrum is observed characteristic of isolated Cu^II^ (Figures 3 c and S12). Modelling the spectra^[26]^ gives *g*_*x*,*y*,*z*_=2.006, 2.078, 2.353 and ^63/65^Cu nuclear hyperfine constants *A*_*x*,*y*,*z*_=50.4, 28, 481.6 MHz (Figure S13) consistent with a square pyramidal geometry at the N‐bound Cu^II^ ion remote from the site of ^*t*^BuOOH binding. The local environment of this Cu^II^ site was further investigated by electron–nuclear double resonance (ENDOR) spectroscopy,^[27]^ which revealed hyperfine interactions with surrounding ^1^H nuclei (Figure 3 d). The spectra were successfully modelled using a point dipole model and taking into account interaction of Cu^II^ with the nearest ^1^H nuclei of the aromatic ligand: that is to the two ‐CH group protons of the pyridine ring (H_py_) and four ‐CH group protons of the phenyl ring (H_ph_) (Figure 3 b). Each ^1^H hyperfine interaction matrix (*A* ^dip^) is defined in its own frame with the *z*‐axis directed along the Cu^II^‐H vector and calculated in the high‐field approximation as *A* ^dip^=[−T; −T; 2 T]. Here, $T=\;\mu_{0}g_{e}g_{n}\mu_{e}\mu_{n}/4\pi r^{3}$ , where *μ*_0_ is the vacuum permeability, *μ*_n_ is the nuclear magneton, *g_n_* is the nuclear g‐factor and *r* is the Cu⋅⋅⋅H distance. Transformation of the *A* ^dip^ tensor into the molecular frame defined by the *g*‐tensor frame of the Cu^II^ site (X∥*g*_xx_, Y∥*g*_yy_, Z∥*g*_zz_) used the Euler angles which were fixed from the SPXRD structure refinement.^[28]^ The *r* values as determined from the orientation selective ENDOR spectra of MFM‐170 treated with ^*t*^BuOOH agree well with the structural data. For example, the obtained Cu^II^⋅⋅⋅H_py_ and Cu^II^⋅⋅⋅H_Ph_ distances of 3.0 Å and 4.0 Å are in good agreement with the values 3.146(2) Å and 4.358(9) Å derived from SPXRD data (Table S4). To characterise the interaction of Cu with ^14^N nuclei, the Larmor frequency of which is much smaller than that of ^1^H, we used hyperfine sublevel correlation (HYSCORE) spectroscopy. The low frequency part of the HYSCORE spectrum of MFM‐170 treated by ^*t*^BuOOH is dominated by cross‐peaks that are assigned to double‐quantum correlation peaks from the ^14^N nucleus (Figure S14). The magnitudes of the ^14^N hyperfine (*A*_iso_=−0.7 MHz) and quadrupole interactions are similar to those reported for pyridines coordinated at the axial position of Cu^II^, for example, in bis‐diketonate complexes.[^29^, ^30^] Thus, ENDOR and HYSCORE data provide clear evidence that the paramagnetic signal observed is due to a Cu^II^ site that is incorporated within the MOF framework. Overall, the experimental data provide good evidence for the proposed reaction cycle and complement the computational study recently reported for a Cu/POM system.^[31]^

The catalytic performance of MFM‐170 was tested with a broad range of substrates using the optimised conditions (Table 2). A comparable yield of α‐tetralone (92 %) was obtained from the oxidation of the chemically similar tetralin. When replacing the α‐carbon to the benzylic position with an oxygen (phthalan), a greater than 99 % yield of phthalide was obtained within 4 h, attributed to the enhanced stabilisation of generated radicals by the adjacent heteroatoms. The oxidation of *p*‐methoxyethylbenzene was more rapid than for ethylbenzene and its halogenated derivatives due to the additional electron‐donating effect of the methoxy group. Substrates with oxidatively sensitive groups, such as ‐OH and ‐CHO groups, were also tested. While the ‐OH group can be retained, ‐CHO undergoes oxidation to ‐COOH species. The reaction proved more active towards diphenylmethane and its halogenated derivatives compared to ethylbenzene and its halogenated derivatives. This was attributed to the increased radical stabilisation offered by increased aromaticity. 9,10‐Dihydroanthracene is more readily oxidised than diphenylmethane due to better resonance stabilisation of the intermediate radical. This arises from the more coplanar orbital overlap resulting in significant over‐oxidation to the diketone leading to a conversion of 98 %, of which 50 % was to the mono‐ketone and 48 % to the di‐ketone. With xanthene, an extremely rapid conversion to xanthone with full oxidation was observed over 3 h consistent with a combination of radical stabilisation effects. While some reported MOFs can catalyse oxidations of small substrates with comparable activity to MFM‐170, larger substrates have been shown to suffer typically from reduced yields due to steric constraints arising from narrow pore diameters.^[15]^ The high porosity of MFM‐170 (cage size up to 22.2×16.3 Å^[17]^) allows more sterically encumbered and complex substrates, such as xanthene (9.18 Å),^[32]^ 9,10‐dihydroanthracene (9.12 Å),^[33]^ fluorene (8.42 Å)^[34]^ and 2,7‐di‐*tert*‐butylfluorene (13.03 Å),^[35]^ to be oxidised catalytically in high yields. Comparison of the activity of MFM‐170 to leading systems places MFM‐170 as a top performing catalyst for benzylic C−H oxidation (Table S5).

**Table 2:** Catalytic oxidation of benzylic C−H compounds to the corresponding ketone using MFM‐170 in the presence of ^*t*^BuOOH.^[a]^ 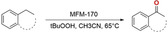

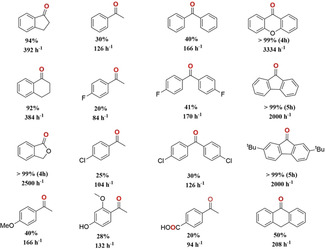

[a] Reaction conditions: substrate (0.25 mmol), MFM‐170 (0.025 mmol), ^*t*^BuOOH (0.75 mmol), ^*t*^BuOOH (0.75 mmol), MeCN (4 mL) 65 °C, 24 h (unless otherwise stated). TOF = 1000×(mol_product_ mol_active Cu_^−1^ h^−1^).

Deactivation of transition metal catalysts often arises via degradation to metal oxide particles.^[15]^ We have demonstrated the utilisation of MFM‐170 and ^*t*^BuOOH in MeCN as an efficient and stable catalyst for benzylic carbon oxidations to give ketones with high selectivity. MFM‐170 can be readily recycled and showed excellent stability over multiple cycles. A viable mechanism for this reaction has been proposed with experimental evidence demonstrating pore‐based catalysis. An in‐depth EPR spectroscopic study, in combination with SPXRD, has provided evidence for the crucial redox behaviour of the [Cu_2_(O_2_CR)_4_] paddlewheels that drives the oxidation. Current work seeks to widen the range of substrates for selective transformations catalysed by designed, heterogeneous coordination polymers.

**Associated content**: Supplementary Information is available in the online version of the paper. Synthetic procedures, characterisation, catalysis testing, and additional analysis of crystal structure. Deposition Number 2017619 (for MFM‐170‐^*t*^BuOOH) contains the supplementary crystallographic data for this paper. These data are provided free of charge by the joint Cambridge Crystallographic Data Centre and Fachinformationszentrum Karlsruhe Access Structures service www.ccdc.cam.ac.uk/structures.

## Conflict of interest

The authors declare no conflict of interest.

## Supporting information

As a service to our authors and readers, this journal provides supporting information supplied by the authors. Such materials are peer reviewed and may be re‐organized for online delivery, but are not copy‐edited or typeset. Technical support issues arising from supporting information (other than missing files) should be addressed to the authors.

Supplementary
