# Supplementary material for: The Origin of Catalytic Benzylic C−H Oxidation over a Redox‐Active Metal–Organic Framework
Source: Angew Chem Int Ed Engl. 2021 Jun 4;60(28):15243–7. doi: 10.1002/anie.202102313 (PMC8361671; doi:10.1002/anie.202102313)
Supplement: Supplementary file 1 — Supplementary [file ANIE-60-15243-s001.pdf]

Angewandte Chemie
Eine Zeitschrift der Gesellschaft Deutscher Chemiker

Supporting Information

The Origin of Catalytic Benzylic C−H Oxidation over a Redox-Active Metal–Organic Framework

Louis Kimberley, Alena M. Sheveleva, Jiangnan Li, Joseph H. Carter, Xinchen Kang, Gemma L. Smith, Xue Han, Sarah J. Day, Chiu C. Tang, Floriana Tuna, Eric J. L. McInnes,* Sihai Yang,* and Martin Schröder**

anie_202102313_sm_miscellaneous_information.pdf

Experimental Section

General

Tritertbutylphosphine (1M solution in toluene), 1,8-diaminonaphthalene, copper(II) nitrate hemipentahydrate, copper(II) chloride, N-tert-butyl-α-phenylnitrone (PBN), phthalan, 4-ethylanisole, bis(4-chlorophenyl)methane (4,4'-DDM), 9,10-dihydroanthracene, xanthene and 2,7-di-tert-butylfluorene were supplied by Sigma Aldrich. 4-Bromophenylboronic acid, 1-fluoro-4-ethylbenzene and bis(4-fluorophenyl)methane were supplied by Fluorochem. $[Pd_2(dba)_3]$, 3,5-dibromopyridine, *t*-butylhydroperoxide (^{t}BuOOH), tetralin, 1-chloro-4-ethylbenzene, diphenylmethane and copper (II) acetate were supplied by Alfa Aesar. Indane, ethylbenzene and fluorine were supplied by Acros Chemicals. All reagents were used without further purification.

Characterisation Techniques

Gas chromatography (GC) was performed using an Agilent 7890B GC system fitted with a HP-5 column and FID detector. ^{1}H and ^{13}C NMR spectra were obtained using a Bruker Avance III 400 MHz spectrometer. Mass spectrometry was carried out using a Waters Acquity UPLC with a Waters SQ detector II for ESI, and an Agilent 1200 series HPLC with an Agilent 6120 quadrupole LC/MS. Elemental analyses were performed using a Thermo Scientific iCAP 6000 Series ICP spectrometer and a Thermo Scientific Flash 2000 organic elemental analyser. TGA was carried out under air at a ramp rate of 5°c min^{-1} using a TA instruments SDT Q600. Samples have been activated by heating at 150 ^{o}C under dynamic vacuum for 12-15 h prior to elemental analysis and TGA experiments. PXRD patterns were collected using a Phillips X'pert diffractometer. N_2 sorption isotherms were collected using a Micromeritics TriStar II Plus gas sorption analyser. SEM images were obtained using a Quanta FEG 650 microscope.

Linker Synthesis

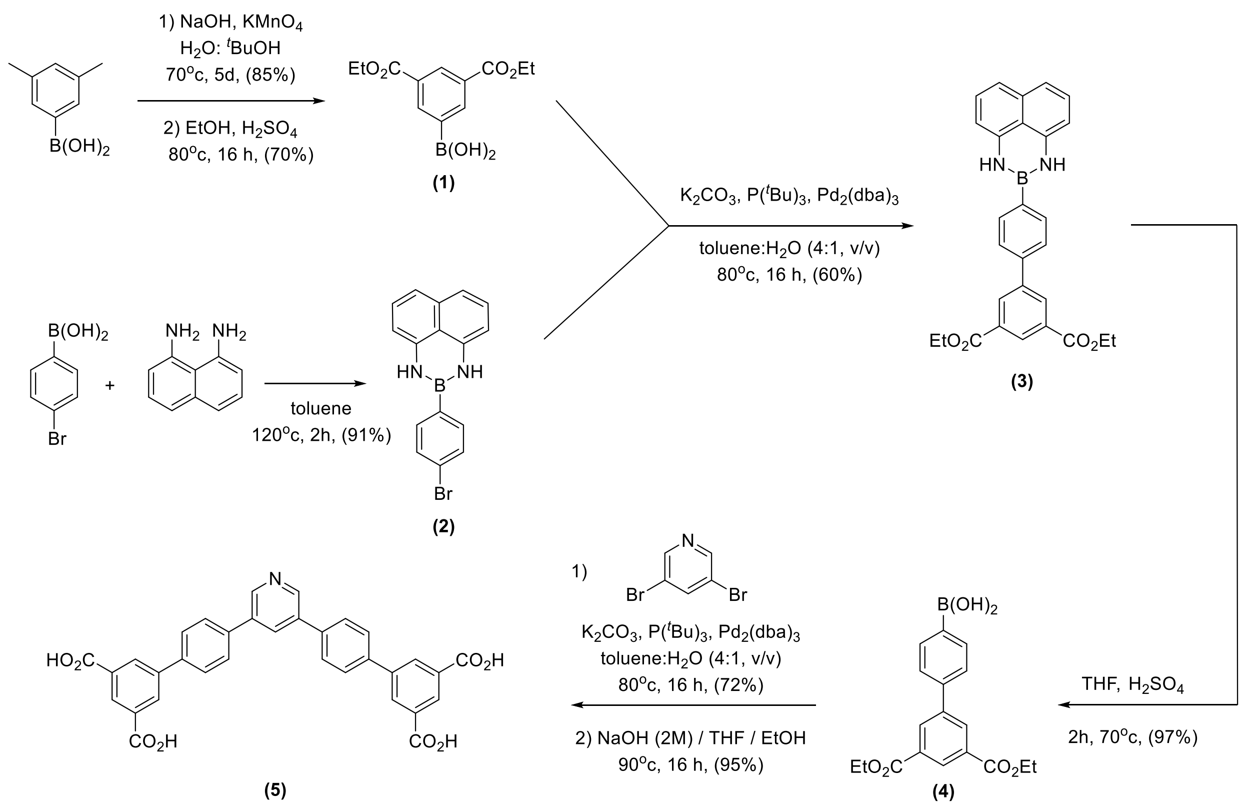

Synthesis of (1,3-diethoxy)phenyl-5-boronic acid ester (1)

3,5-Dimethylboronic acid (25 g, 0.17 mol) and NaOH (24 g, 0.6 mol) were dissolved in a mixture of H_2O: ${}^tBuOH$ (1 L, 4:1 *v/v*) and heated to 50 °C. $KMnO_4$ (165 g, 1 mol) was added to the reaction over the course of 2 d and the temperature increased to 70 °C after two thirds were added. Excess $KMnO_4$ was reduced by adding ${}^iPrOH$ (150 ml) and refluxing for 1 h. The precipitated MnO_2 was removed by filteration and washed with boiling water. The solvent was reduced *in vacuo* and the white solid product precipitated by the addition of conc. HCl. (29.95g, 85%). ^1H NMR (400 MHz, DMSO-d_6) δ ppm: 8.43 (2H, s br), 8.50 (1H, s), 8.61 (2H, s), 13.17 (2H, s) ^{13}C NMR (100 MHz, DMSO-d_6) δ ppm: 130.16, 131.40, 138.91, 166.78. MS (ESI) *m/z*: 211.0256 $(M+H)^+$. Elemental analysis (calc./obs., %): C 45.77/45.55, H 3.36/3.30.

The product was refluxed at 80 °C in a solution of EtOH (500 ml) and H_2SO_4 (10.5 ml) overnight. The solvent was reduced *in vacuo*. and the white solid product precipitated by the addition of cold water (26.54 g, 70%). ^1H NMR (400 MHz, DMSO-d_6) δ ppm: 1.35 (6H, t, J = 7.08 Hz), 4.37 (4H, q, J = 7.08 Hz) 8.50 (1H, s), 8.63 (2H, s). ^{13}C NMR (100 MHz, DMSO-d_6) δ ppm: 14.21, 61.13, 129.68, 131.09, 139.12, 165.32. MS (ESI) *m/z*: 267.10 $(M+H)^+$. Elemental analysis (calc./obs., %): C 54.17/53.93, H 5.68/5.61.

Synthesis of diaminonaphthalene protected (4-bromophenyl) boronic acid (2)

4-Bromophenylboronic acid (3.11 g, 15.5 mmol) and 1,8-diaminonaphthalene (2.69 g, 17 mmol) were heated at 120 °C in toluene (250 ml) for 2 h. The solvent was removed *in vacuo* and the product recrystallized from CH_2Cl_2/petroleum ether as a brown solid (4.56 g, 91%). ^1H NMR (400 MHz, DMSO-d_6) δ ppm: 5.99 (2H, s,

br), 6.43 (2H, d, *J* = 7.09 Hz) 7.08 (2H, d, *J* = 8.08 Hz), 7.16 (2H, m), 7.54 (2H, m), 7.59 (2H, m). ^{13}C NMR (100 MHz, DMSO-d_6) δ ppm: 106.15, 118.15, 124.90, 127.61, 131.44, 133.01, 136.29, 140.75. MS (ESI) *m/z*: 321.0204 (M-H)$^-$. Elemental analysis (calc./obs., %): C 59.50/59.15, H 3.74/3.71, N 8.67/8.50, Br 24.74/24.97.

Synthesis of 1,3-diethyl 5-(4-{2,4-diaza-3-boratricyclo[7.3.1.0^{5,13}]trideca-1(12),5,7,9(13),10-pentaen-3-yl}phenyl)benzene-1,3-dicarboxylate (3)

1 (4.65 g, 17.48 mmol), **2** (3.77 g, 11.67 mmol) and K_2CO_3 (5 g, 36 mmol) were dissolved in a toluene:water mixture (500 ml, 4:1 *v/v*) and degassed for 1 h at 60 °C. [$Pd_2(dba)_3$] (1 g, 1.1 mmol) and ^{t}Bu_3P (1M solution in toluene, 4.33 ml, 4.33 mmol) were added, the temperature increased to 80 °C and the reaction stirred overnight. The solution was filtered through Celite® to remove any reduced Pd^0 species. The product was extracted into CH_2Cl_2 and the solution dried with $MgSO_4$ before being recrystallized as a yellow solid from CH_2Cl_2/petroleum ether (3.23 g, 60%). ^{1}H NMR (400 MHz, DMSO-d_6) δ ppm: 1.38 (6H, t, *J* = 7.09 Hz), 4.41, (4H, q, *J* = 7.09 Hz), 6.62 (2H, d, *J* = 6.85 Hz), 6.92 (2H, d, *J* = 7.08 Hz), 7.10 (2H, t, *J* = 7.83 Hz), 7.84 (2H, d, *J* = 8.31 Hz), 8.10 (2H, d, *J* = 8.07 Hz), 8.38 (2H, s), 8.47 (3H, s). ^{13}C NMR (100 MHz, DMSO-d_6) δ ppm: 13.77, 61.02, 105.30, 125.81, 127.26, 130.91, 133.22, 141.90, 164.47. MS (ESI) *m/z*: 503.1529 (M+K)$^+$. Elemental analysis (calc./obs., %): C 72.43/72.00, H 5.43/5.41, N 6.03/5.91.

Synthesis of {4-[3,5-bis(ethoxycarbonyl)phenyl]phenyl}boronic acid (4)

3 (3.2 g, 6.89 mmol) was dissolved in a THF (300 ml) and H_2SO_4 (2 M, 50 ml) mixture and heated at 80 °C for 1h. Precipitated salts were removed by filtration and the filtrate heated at 80 °C for a further 1.5 h. The solvent was reduced *in vacuo* and the product precipitated as a light pink solid on addition of cold water (2.30 g, 97%). ^{1}H NMR (400 MHz, DMSO-d_6) δ ppm: 1.38 (6H, t, *J* = 7.09 Hz), 4.39, (4H, q, *J* = 7.09 Hz), 7.72 (2H, d, *J* = 7.82 Hz), 7.93 (2H, d, *J* = 7.82 Hz), 8.17 (2H, s br), 8.42 (2H, s br) 8.45 (1H, s br). ^{13}C NMR (100 MHz, DMSO-d_6) δ ppm: 14.79, 62.04, 126.57, 128.95, 132.03, 135.70, 142.05, 144.34, 165.48. MS (ESI) *m/z*: 343.1204 (M-H)$^-$. Elemental analysis (calc./obs., %): C 63.19/63.38, H 5.60/5.66.

Synthesis of H_4PPYD (5)

4 (2.35 g, 6.57 mmol), 3,5-dibromopyridine (0.75g, 3.16 mmol) and K_2CO_3 (1.25 g, 9.07 mmol) were dissolved in a toluene:water mixture (225 ml, 4:1 *v/v*) and degassed for 1 h at 60 °C. [$Pd_2(dba)_3$] (0.3 g, 0.33 mmol) and ^{t}Bu_3P (1M solution in toluene, 1.30 ml, 1.30 mmol) were added, the temperature increased to 80 °C and the reaction stirred overnight. The solution was then filtered through Celite® to remove any reduced Pd^0 species. The product was extracted into CH_2Cl_2 and the solution dried with $MgSO_4$ before being recrystallized as a white solid from CH_2Cl_2/MeOH. (1.54 g, 72%). ^{1}H NMR (400 MHz, $CDCl_3$) δ ppm: 1.46 (12H, t, *J* = 7.34 Hz), 4.46 (8H, q, *J* = 7.34 Hz), 7.82 (8H, m), 8.17 (1H, s br), 8.54 (4H, s br), 8.70 (2H, s br), 8.92 (2H, s br). ^{13}C NMR (100 MHz, $CDCl_3$) δ ppm: 14.05, 61.22, 127.56, 127.66, 129.22, 131.30, 131.78, 137.16, 138.81, 140.66, 165.45 MS (APCI) *m/z*: 672.2587 (M+H)$^+$. Elemental analysis (calc./obs., %): C 73.31/67.30, H 5.55/5.57, N 2.09/2.07.

The product was stirred at 90 °C in a solution of THF (37.5 ml), EtOH (37.5 ml) and NaOH (2 M, 75 ml) overnight. The organic components were then evaporated, and the product precipitated as a white solid by decreasing the pH to ~2 through the careful addition of 2M HCl. The fine product was washed with water multiple times and dried (1.18 g, 95%). ^{1}H NMR (400 MHz, DMSO-d_6) δ ppm: 7.90 (4H, d, J = 8.53 Hz), 8.04 (4H, d, J = 8.28 Hz), 8.42 (4H, d, J = 1.25 Hz), 8.46 (1H, t, J = 2.26 Hz), 8.49 (2H, t, J = 1.51 Hz), 8.98 (2H, d, J = 2.01 Hz). ^{13}C NMR (100 MHz, DMSO-d_6) δ ppm: 126.54, 129.32, 131.45, 132.26, 136.57, 138.89, 140.20, 141.68, 166.24 MS (ESI) *m/z*: 560.13 $(M+H)^+$. Elemental analysis (calc./obs., %): C 70.84/68.54, H 3.78/3.63, 2.50/2.15.

Synthesis of MFM-170

5 (192 mg, 0.36 mmol) and $Cu(NO_3)_2\cdot2.5H_2O$ (298 mg, 1.28 mmol) were dissolved in a solution of DMF:H_2O (48 ml, 5:1 *v/v*) and HNO_3 (0.3 ml). The solution was heated to 80 °C without stirring overnight to yield MFM-170 as blue crystals. The product was filtered and washed with hot DMF to remove any unreacted linker. Elemental analysis (calc./obs., %): C 54.84/53.42, H 4.38/4.30, N 6.09/5.91, Cu 13.82/13.25.

Procedure for Catalytic Benzylic Oxidations

A solution of benzylic substrate (0.25 mmol), MFM-170 (25 mg, 0.022 mmol) and ^{t}BuOOH (100 µl) in MeCN (4 mL) were stirred vigorously at 65ºC. At the end of the reaction the catalyst was separated by centrifugation and recycled, as required. GC analysis was carried out on the reaction solution to determine % yields using a combination of internal and external standards.

Synchrotron powder X-ray diffraction of ^{t}BuOOH -Loaded MFM-170

A freshly synthesised sample of MFM-170 was exchanged with acetone over the course of 1 week before being dried *in vacuo* and loaded into a 0.7 mm borosilicate capillary. The loaded capillary was placed in a Schlenk flask and heated to 150 °C under active vacuum for *c.a.* 16 h. Activation of the sample was visualised by a colour change of from light blue to purple. ^{t}BuOOH vapour was introduced by heating a small amount of liquid ^{t}BuOOH stabilised in decane to 100 °C in a separate Schlenk flask, and allowing the vapour to enter the flask containing the capillary *via* a three way valve. The system was allowed equilibrate for *c.a.* 5 h, the capillary was removed and rapidly sealed.

Synchrotron powder X-ray diffraction scans were carried out on the I11 high-resolution powder diffraction beamline at Diamond Light Source (UK). Data were collected between 0 and 150° using a step size of 0.001° with five multi-analysing crystal (MAC) detectors. Rietveld refinement of the structure was carried out on the data between 1.7 and 25° using the TOPAS software package. Atomic parameters for the reported structure of MFM-170 (CCDC no. 1853512) were used as a starting point before adding successive ^{t}BuOOH molecules (hydrogens omitted) into the framework as semi-rigid bodies. Approximate positions of the ^{t}BuOOH molecules were found using the simulated annealing approach before further refinement was used to find the optimal orientation of the guest molecules. Accuracy of the final model was verified by the convergence of the

weighted profile factor (R_{wp}), the chemical sense of the model and the good correlation between the observed and calculated diffraction patterns (Figure S8). Crystal data of MFM-170-^{t}BuOOH is deposited at Cambridge Crystallographic Data Centre (CCDC) 2017619.

EPR Spectroscopy

Continuous-wave Electron Paramagnetic Resonance (EPR) measurements were carried out at X-band (~9.4 GHz) microwave frequency using a Bruker EMX-Micro spectrometer equipped with a SHQH-HS-4123D2 resonator. EPR spectra for the spin trap experiment were recorded with a modulation amplitude of 1 G, microwave frequency of 9.4 GHz and a microwave power of 6.3 mW.

Spin trapping experiments were performed according to the following general procedure: a solution of N-tert-butyl-α-phenylnitrone (PBN) (0.4 mol L^{-1}) in dry deoxygenated MeCN was prepared prior to the measurements and kept in a freezer at -20 ^{o}C. Experiments were conducted in one of two ways:

Method 1: The PBN solution (0.1 mL) was mixed with 0.1 mL of solution of a given sample in MeCN (^{t}BuOOH, MFM-170 or mixture of ^{t}BuOOH and MFM-170). In each case, the resulting mixture was heated to 65 ^{o}C and stirred for 2 min prior to being transferred to a glass capillary (1.6 mm od), and spectra recorded at room temperature. Estimation of the efficiency of PBN adduct formation was achieved through the analysis of the second integral of the resultant spectra.

Method 2 (*in situ* experiments): A solution of PBN (0.1 mL) was mixed with a solution (0.1 mL) containing a mixture of ^{t}BuOOH and MFM-170 in MeCN (ratios as for the optimized oxidation procedure). The solution was sampled in a glass capillary (1.6 mm od), placed in the spectrometer equipped with a cryostat, stabilized at 65 ^{o}C and measured immediately (the duration of the measurement was 96 sec).

Low-temperature (10 K) EPR spectra of MFM-170 and MFM-170 mixed with ^{t}BuOOH were recorded on a Bruker EMX 300 EPR spectrometer equipped with a high-Q X-band (ca. 9.4 GHz) resonator and a liquid He cryostat. Field corrections were applied by measuring relevant EPR standards (Bruker strong pitch and DPPH). For accuracy, the tube size and tube position in the cavity were kept constant.

Pulsed electron paramagnetic resonance (EPR) measurements of powdered samples of MFM-170 and MFM-170 treated with ^{t}BuOOH were performed using an X-band (*ca.* 9.7 GHz) microwave frequency on a Bruker Elexsys E580 spectrometer. The microwave frequency was measured with a built-in digital counter and the magnetic field was calibrated using a Bruker strong pitch reference sample. Echo-detected (ED) spectra were measured at X-band using sequence ($\pi/2 - \tau - \pi - \tau$ – echo), where $\pi/2$ and π are 16 ns and 32 ns respectively. The interpulse τ delay was 150 ns. Electron-nuclear double resonance (ENDOR) measurements used the Davies sequence (π_{inv} − RF − $\pi/2 - \tau - \pi - \tau$ – echo) with microwave inversion and radiofrequency (RF) π-pulse durations of 200 and 1200 ns, respectively.[1] HYSCORE spectra were measured at X-band using a standard pulse sequence ($\pi/2 - \tau - \pi/2 - T_1 - \pi - T_2 - \pi/2 - \tau$ – echo) the length of microwave pulses were $\pi/2$ = 16 ns and π=22 ns.[2] The time delay between the first two pulses was taken as τ = 136 ns and τ = 200 ns,

values of T_1 and T_2 times were $T_1=T_2=70$ *ns*. The (128x128) HYSCORE data array was recorded with the time increment of 20 ns, then two-dimensional Fourier transform (FT) magnitude spectra were calculated. The FT spectra were summed to avoid distortions due to the blind spot effect.

Theoretical modeling of all EPR data was performed using EasySpin toolbox (Version 5.2.27) for Matlab.

Supplementary Figures and Tables

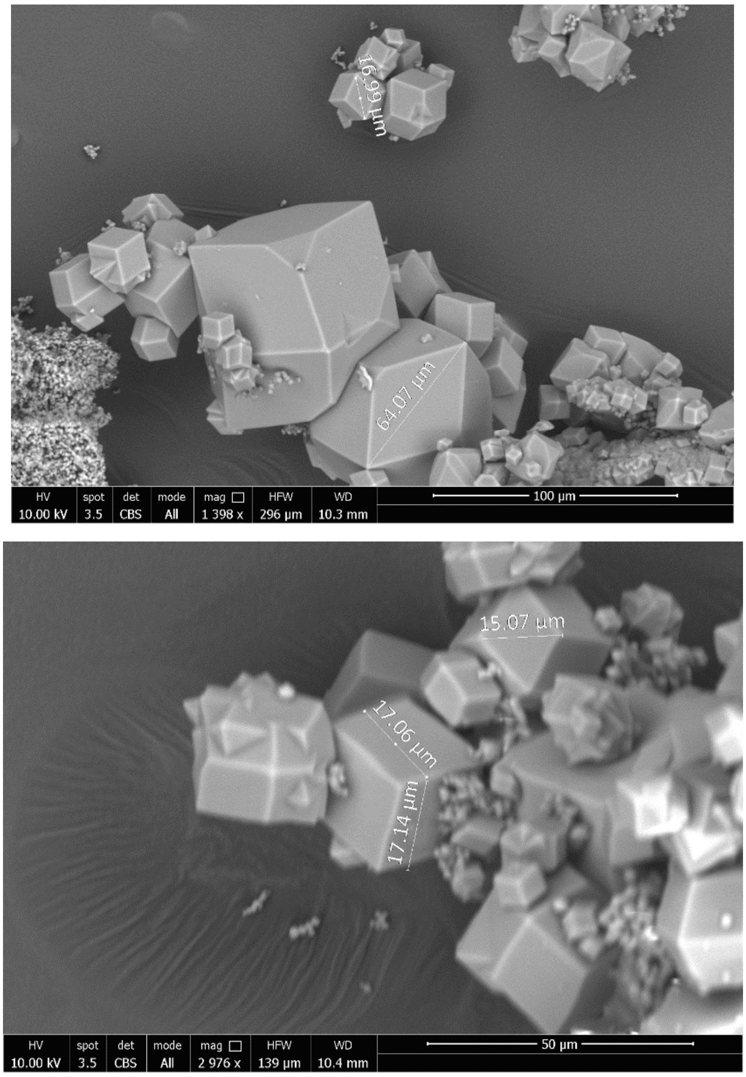

Figure S1. SEM images of pristine MFM-170.

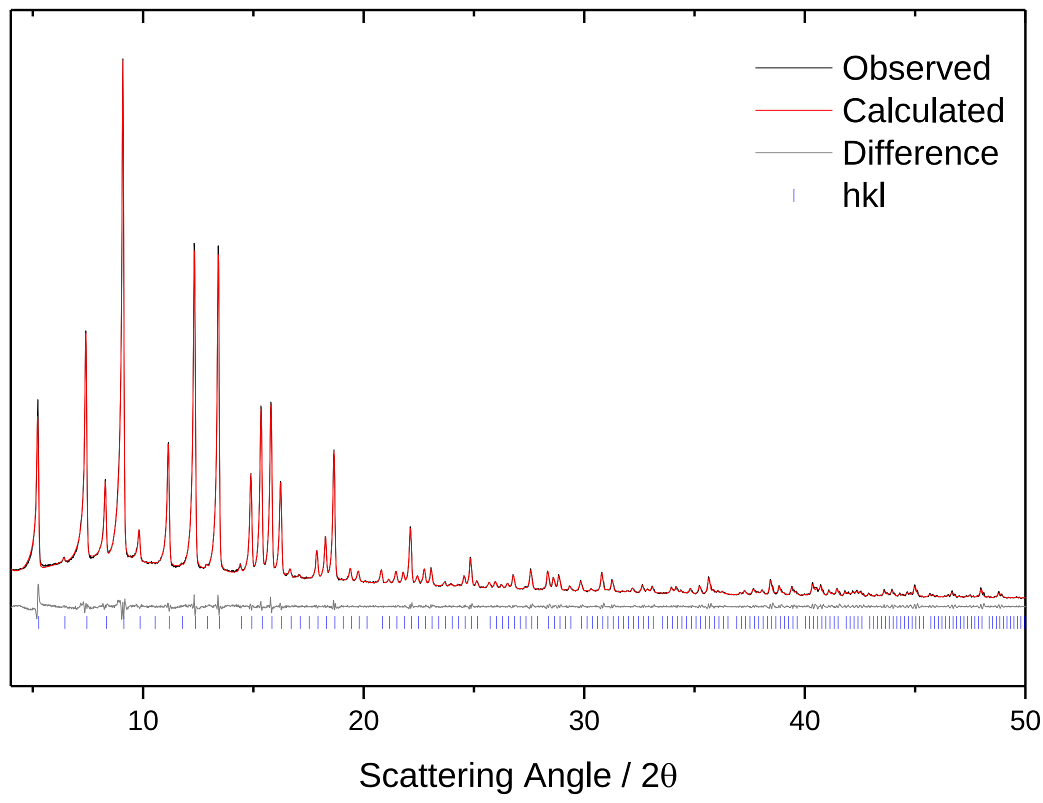

Figure S2. Fitted profile for Pawley refinement of MFM-170, gof = 2.39, Rwp = 3.60 %, Rp= 2.57%.

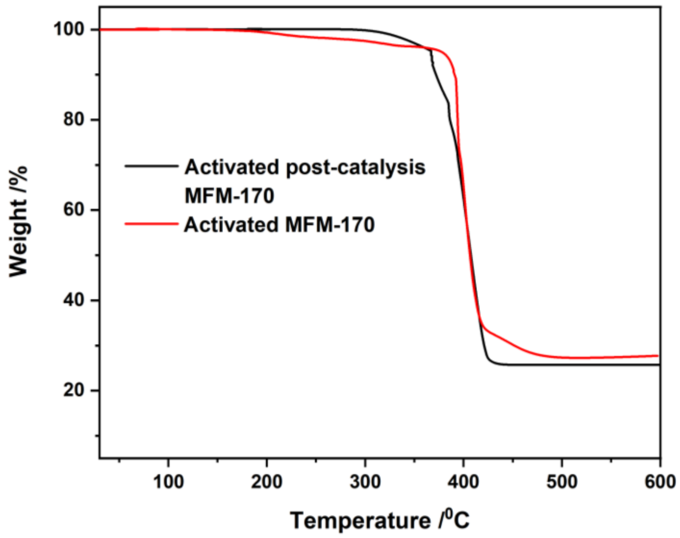

Figure S3. TGA plots of fresh and recycled MFM-170 samples, which were activated before the TGA tests.

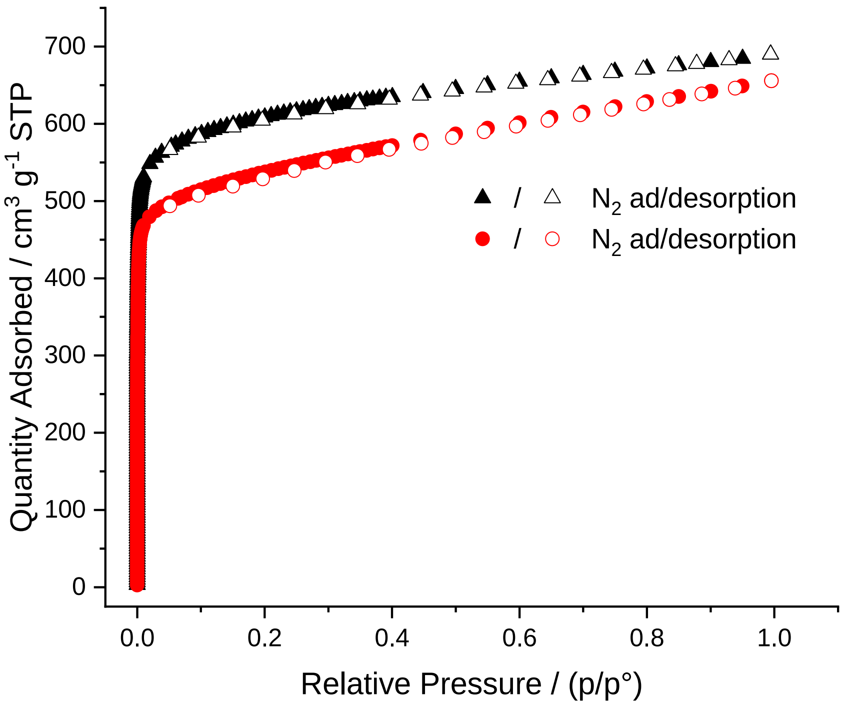

Figure S4. Adsorption isothem for N_2 in fresh MFM-170 (black) and recycled MFM-170 (red) measured at 77 K. The apparent surface area is 2325 and 2076 m^2 g^{-1}, respectively.

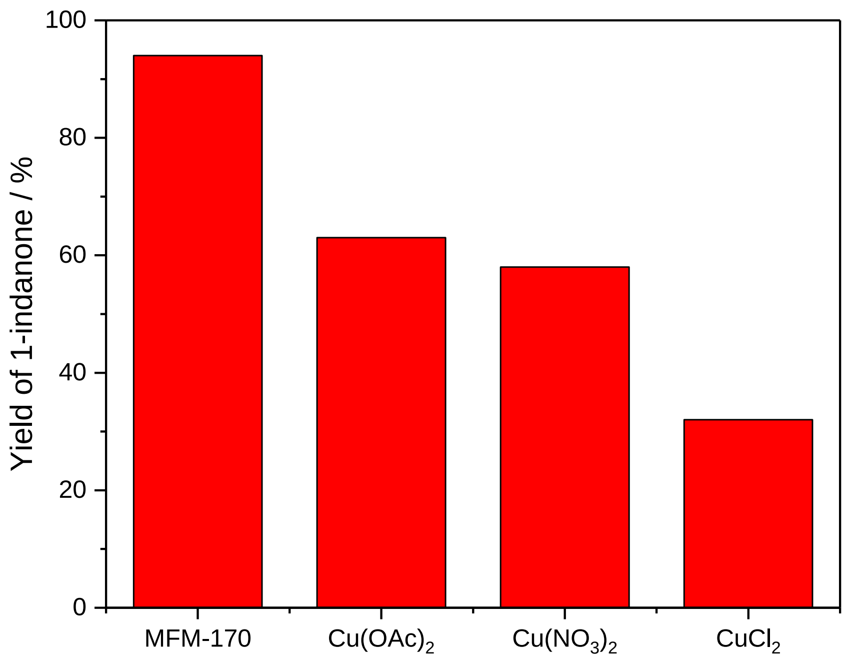

Figure S5. Yields of 1-indanone obtained from catalytic reactions with MFM-170 and various homogeneous Cu(II) salts. Reaction conditions: indane (0.25 mmol), catalyst (0.025 mmol), ^{t}BuOOH (0.75 mmol) MeCN (4 mL) 65 °C, 24 h.

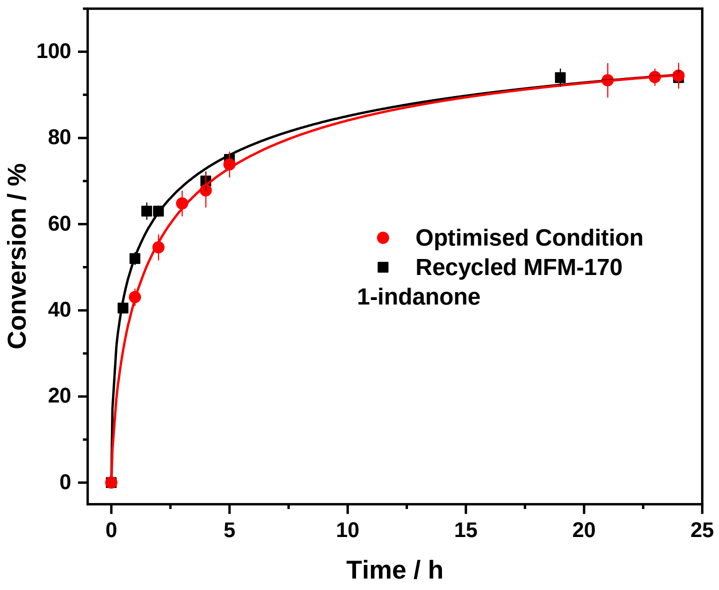

Figure S6. Plot of time *vs* conversion for the oxidation of indane catalysed by recycled MFM-170 (black) and fresh MFM-170 (red), showing similar conversion rates. Reaction conditions: indane (0.25 mmol), MFM-170 (0.025 mmol, 0.05 mmol Cu), ^{t}BuOOH (0.75 mmol), MeCN (4 mL) 65 °C, 24 h.

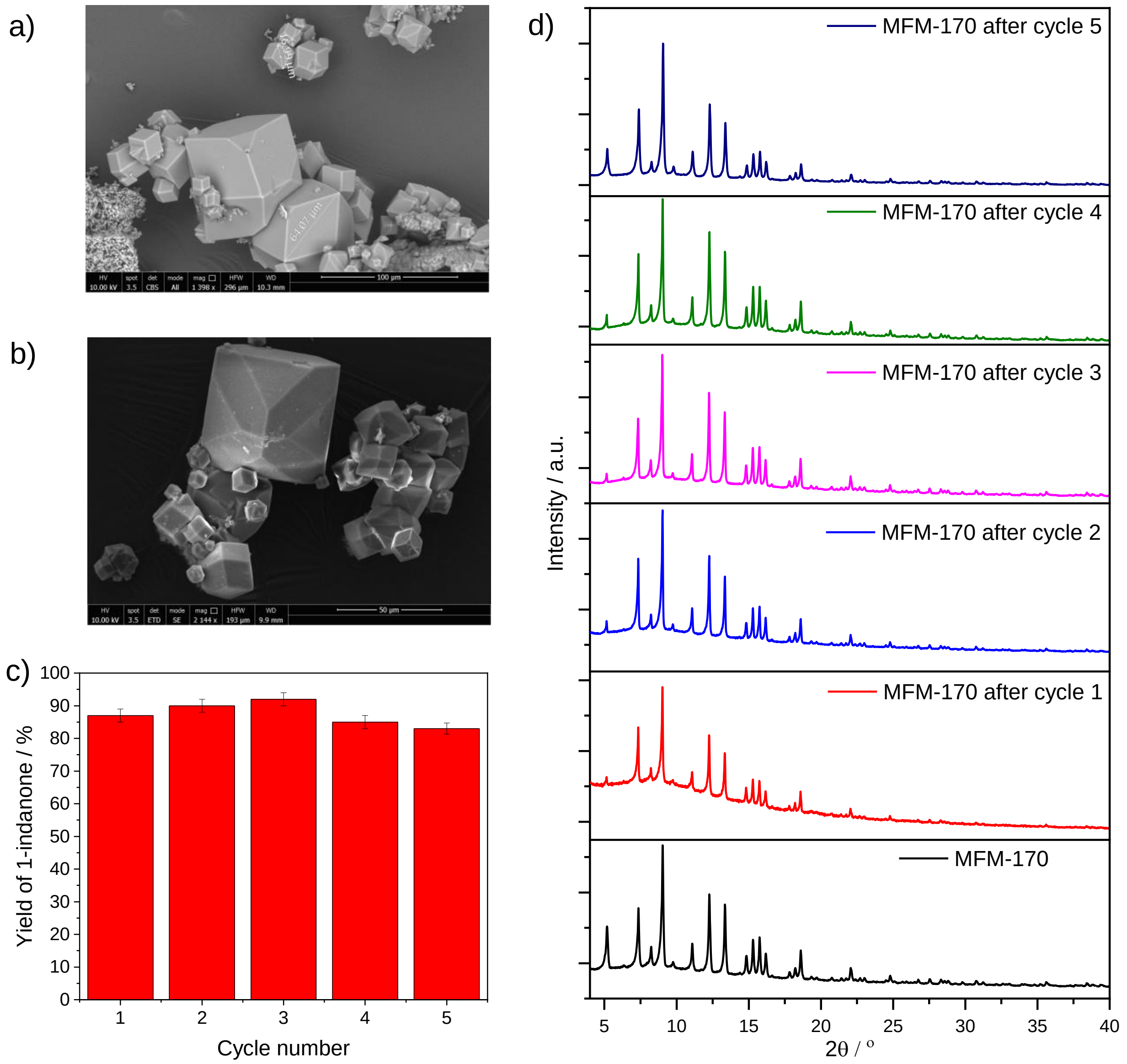

Figure S7. SEM images of MFM-170 (a) before and (b) after 5 cycles. (c) Yields of 1-indanone after each cycle. The overall variation of <10 % across all runs are within experimental error considering the relatively small scale of each reaction. (d) PXRD patterns for MFM-170 after each consecutive cycle of catalysis.

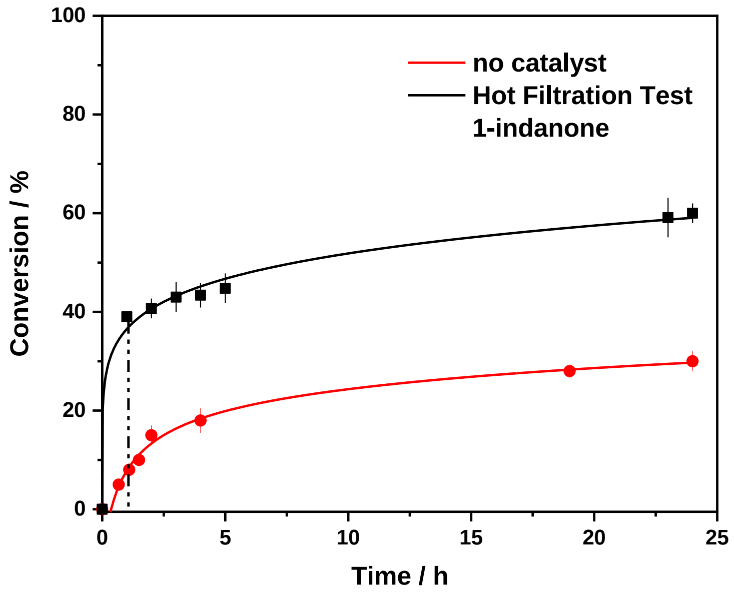

Figure S8. Plot of time *vs* conversion for the indane oxidation without catalyst (red) and hot filtration (leach) test (black). The catalyst was removed at the dash line in the black plot. The further conversion upon removal of the catalyst is likely due to the thermal activation of the oxidant. Reaction conditions: indane (0.25 mmol), MFM-170 (0.025 mmol, 0.05 mmol Cu), ^{t}BuOOH (0.75 mmol), MeCN (4 ml) 65 ºC, 24 h.

Table S1. Elemental analysis on MFM-170 samples

	C/%	H/%	N/%	Cu/%
MFM-170 (Calculated)	56.6	2.7	2.0	18.1
MFM-170 (Activated)	54.5	2.8	2.1	18.2
Recycled MFM-170	55.6	3.0	1.9	17.5

The results are subject to an absolute error of ±0.3-0.5%. Only a trace amount of Cu (within the detection limit of 0.5%) was detected in the reaction solution after removal of the MFM-170 catalyst.

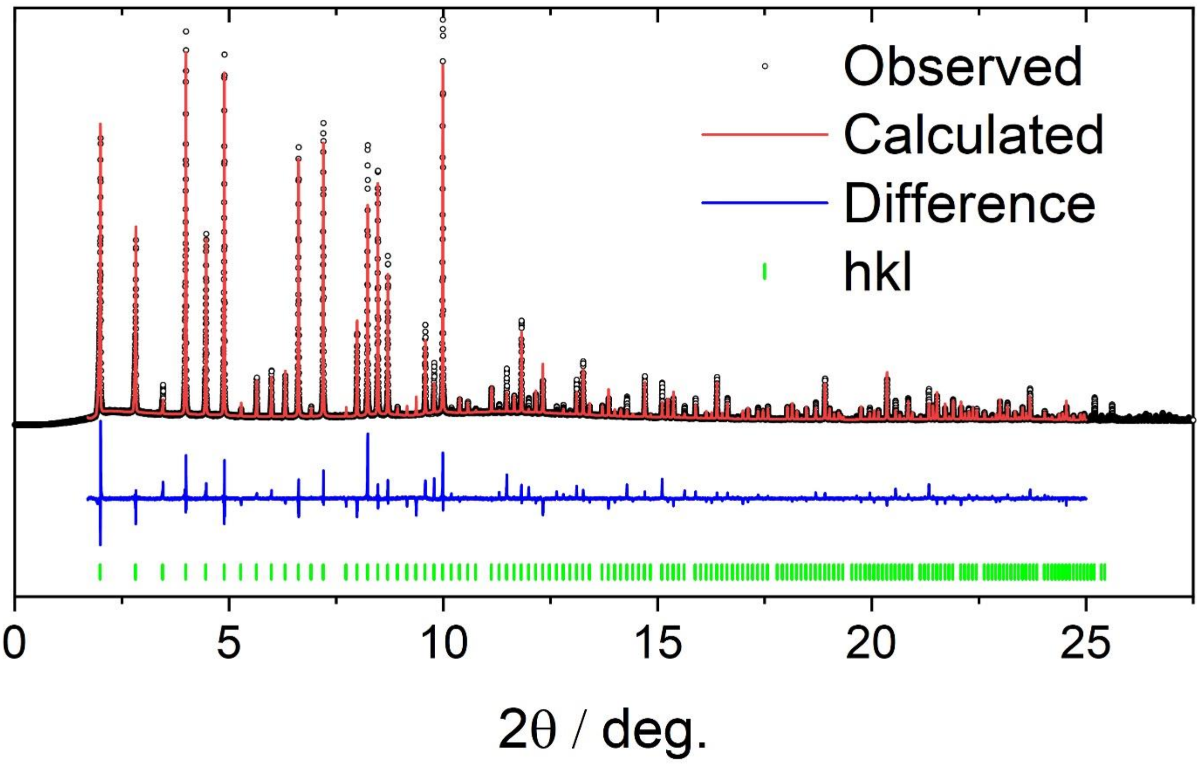

Figure S9. Fit profile for Rietveld refinement of ^{t}BuOOH-loaded MFM-170, gof = 3.33, Rwp = 9.96 %. R_p= 6.49 % R_{bragg}= 6.62%.

Table S2. Parameters used for and the results of the bond valence sum calculations.

$$BVS_{(Cu)} = \sum exp \frac{(R_0 - R)}{B}$$

MFM-170				
Oxidation state	R_0 / Å[a]	B / Å[a]	Cu-O bond length / Å	$BVS_{(Cu)}$
+1	1.601	0.335		1.39
+2	1.679	0.36	1.955	1.86
+3	1.735	0.37		2.21

MFM-170-*t*BuOOH				
Oxidation state	R_0 / Å[a]	B / Å[a]	Cu-O bond length / Å	$BVS_{(Cu)}$
+1	1.601	0.335		0.99
+2	1.679	0.36	2.070	1.35
+3	1.735	0.37		1.62

[a]obtained from ref 3-5.

Table S3. Parameters for the simulations of EPR spectra.

MeCN/PBN/ ^{t}BuOOH/MFM-170 at 65ºC						
Weight	Adduct	g-factor	$A^{14}N$ / G	$A^{\beta}H$ / G	Linewidth/mT	Ref.
0.8	*t*-BuO$^{\bullet}$	2.0064	14.25	2.25	0.31	6,7
0.2	*t*-BuOO$^{\bullet}$	2.0064	13.47	1.4	0.25	8

Table S4. Simulation parameters of the ENDOR spectra.

	ENDOR determined Cu(II)…^{1}H distance, *r* / Å	Structural refinement Cu(II)…^{1}H distance, *r* / Å	β Euler angle/ degrees
H_{py}	3.0	3.146(2)	140
H_{ph}	4.0	4.358(9)	99.8

The values of β, the Euler angle, were evaluated from structural refinement data; the values of the angles α and γ had no impact on the ENDOR spectra and therefore are not shown. Lorentzian linewidth equals 0.2 MHz for all samples.

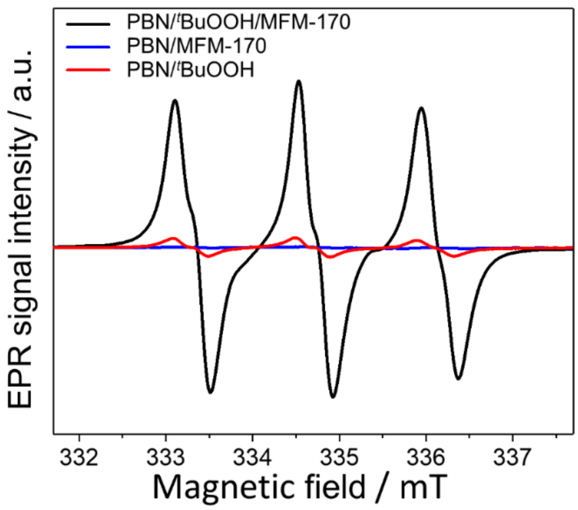

Figure S10. Spin-trapping X-band (9.4 GHz) EPR spectra at 298 K for MeCN solutions of PBN/^{t}BuOOH/MFM-170 (black), PBN/ ^{t}BuOOH (blue) and PBN/MFM-170 (red, measured under similar experimental conditions). Comparison of the double integral of the spectra indicate a 6- and 130-fold reduction in signal intensity for the latter two spectra, respectively, compared to the first. The asymmetric shape is due to restricted motion from anisotropic effects.[9]

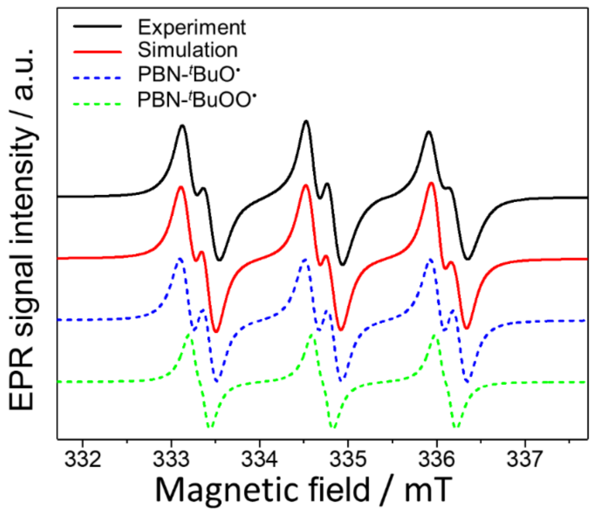

Figure S11. EPR X-band (9.4 GHz) EPR spectrum of PBN spin trap with ^{t}BuOOH and MFM-170 in MeCN at 338 K (black). Simulated spectrum (red) is the combined spectrum of PBN-^{t}BuO· radical (blue; a_H = 2.25 G; a_N = 14.25 G) and PBN-^{t}BuOO· radical (green ; a_H = 1.4 G; a_N = 13.47 G). The presence of the PBN-^{t}BuOO· adduct was required to achieve better agreement between experimental and simulated spectrum.

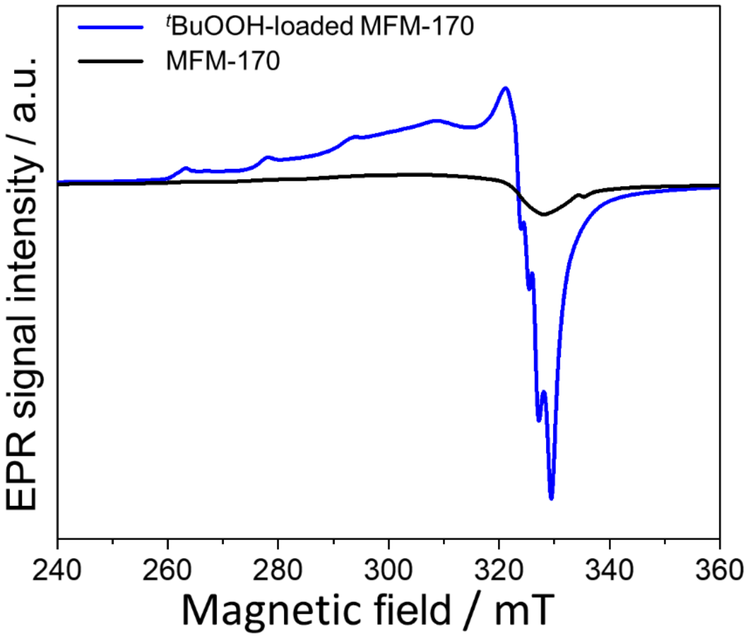

Figure S12. CW X-band (9.4 GHz) EPR spectra at 10 K of MFM-170 (black) and MFM-170 with ^{t}BuOOH

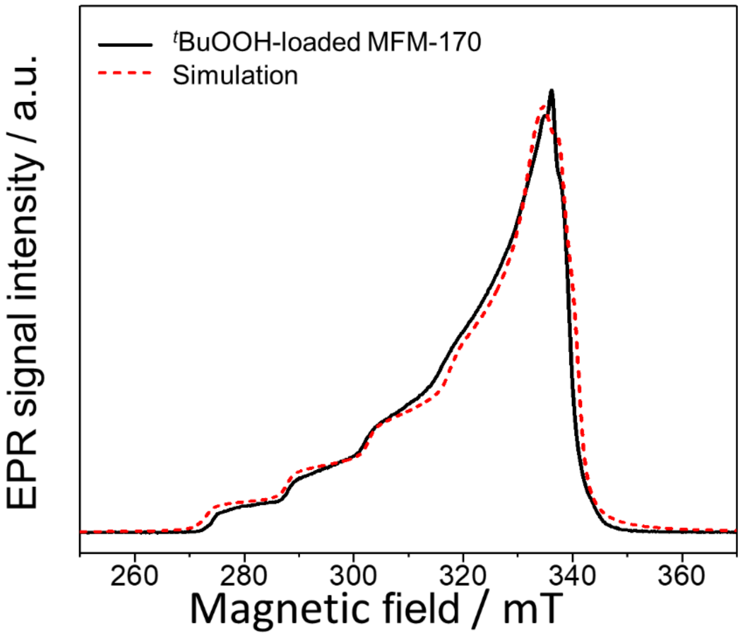

Figure S13. Experimental (black) and simulated (red) echo-detected (ED) X-band spectra (9.724 GHz) at 5 K of MFM-170 treated with ^{t}BuOOH. Simulation parameters: g_x = 2.0058, g_y = 2.078, g_z = 2.353 and $^{63/65}$Cu nuclear hyperfine interactions (nuclear spin, I = 3/2) of A_x = 50.4, A_y = 28, A_x = 481.6 MHz.

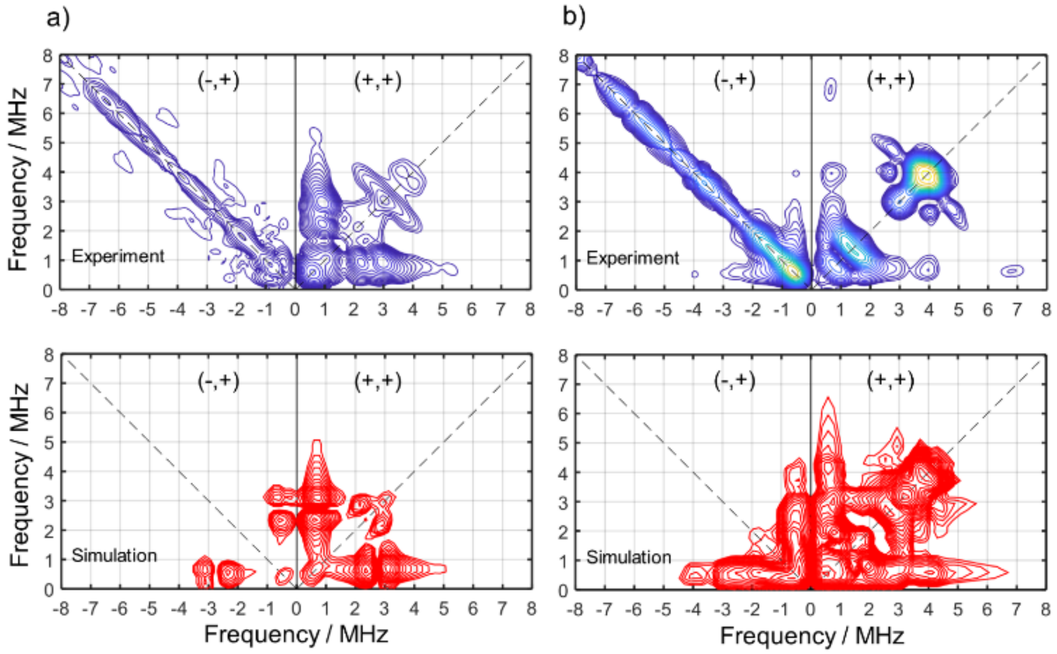

Figure S14. Top: X-band (9.724 GHz) HYSCORE spectra at magnetic field positions (a) 290 mT and (b) 338 mT measured at 5 K for ^{t}BuOOH-loaded MFM-170. Bottom: corresponding simulations with ^{14}N hyperfine and quadrupole interaction parameters: A = [-0.4,-0.4,-1.3] MHz with Euler angles [α, β, γ] = [0, 0, 0] and $|e^2qQ/h|$ = 4.1 MHz with Euler angles [α, β, γ] = [0, 0, 0] and η = 0.3.

Table S5. Literature examples of catalytic benzylic C-H oxidation.

Entry	Catalyst	Oxidant	Additional Reagents	Reaction time/ h	Temperature / °C	Substrate	Yield	Ref.
1	MFM-170	^tBuOOH	-	24	65	indane	94	This work
2	MFM-170	^tBuOOH	-	24	65	tetralin	92	This work
3	MFM-170	^tBuOOH	-	24	65	ethylbenzene	20	This work
4	MFM-170	^tBuOOH	-	24	65	diphenylmethane	40	This work
5	MFM-170	^tBuOOH	-	3	65	xanthene	>99	This work
6	MFM-170	^tBuOOH	-	5	65	fluorene	>99	This work
7	$FeCl_3$	^tBuOOH	pyridine	24	82	indane	61	9
8	$FeCl_3$	^tBuOOH	pyridine	24	82	tetralin	41	9
9	$FeCl_3$	^tBuOOH	pyridine	24	82	diphenylmethane	86	9
10	$FeCl_3$	^tBuOOH	pyridine	24	82	ethylbenzene	17	9
11	β-FeOOH	^tBuOOH	-	8	70	xanthene	>99	10
12	Fe(BTC)	^tBuOOH	-	24	70	Indane	31	11
13	Fe(BTC)	^tBuOOH	-	24	70	tetralin	62	11
14	Fe(BTC)	^tBuOOH	-	24	70	xanthene	>99	11
15	Ni-MOF-74	^tBuOOH	[bmim]Br	12	25	indane	84	12
16	Ni-MOF-74	^tBuOOH	[bmim]Br	12	25	tetralin	75	12
17	Ni-MOF-5	O_2	-	6	150	ethylbenzene	21	13
18	Ru/NHC-bipy	^tBuOOH	-	3	70	diphenylmethane	20	14
19	HKUST-1	O_2	NHPI	9	60	fluorene	39	15
20	Cu-$CuFe_2O_4$/ HKUST-1	O_2	NHPI	9	60	fluorene	>99	15
21	Cp_2VCl_2	^tBuOOH	-	120	30	ethylbenzene	90	16
22	Cp_2VCl_2	^tBuOOH	-	120	30	diphenylmethane	95	16

References

1 E. R. Davies, *Phys. Lett. A* **1974**, *47*, 1–2.

2 P. Höfer, A. Grupp, H. Nebenführ and M. Mehring, *Chem. Phys. Lett*. **1986**, *132*, 279–282.

3 O.C Gagne and F.C. Hawthorne, *Acta Crystallogr. B. Struct. Sci. Cryst. Eng. Mater*. **2015**, *71*, 562-578.

4 S.V. Krivovechev, *Z. Kristallogr*. **2012**, *227*, 575-579.

5 S. Mahapatra, J.A. Halfen, E.C. Wilkinson, G. Pan, X. Wang, V.G. Young, C.J. Cramer, L. Que, and W.B. Tolman, *J. Am. Chem. Soc*. **1996**, *118*, 11555-11574.

6 E. G. Janzen and C. A. Evans, *J. Am. Chem. Soc*. **1973**, *95*, 8205–8206.

7 L. D. Haire, P. H. Krygsman, E. G. Janzen and U. M. Oehler, *J. Org. Chem*. **1988**, *53,* 4535–4542.

8 J. Keizer, *J. Am. Chem. Soc*. **1983**, *105*, 1494–1498

9 M. Nakanishi and C. Bolm, *Adv. Synth. Catal.* **2007**, *349*, 861 – 864

10 X. Kang, X. Sun, Q. Zhu, X. Ma, H. Liu and B. Han, *ChemComm*. **2016**, *52*, 4687–4690.

11 A. Dhakshinamoorthy, M. Alvaro and H. Garcia, *J. Catal.* **2009**, *267*, 1-4.

12 C. Guo, Y. Zhang, Y. Zhang and J. Wang, *ChemComm.* **2018**, *54*, 3701-3704.

13 M.M Peng, U. Jin, J.M. Ganesh, A. Aziz, R. Vinodh, M. Palanichamy, and H.T. Jang, *Bull. Korean Chem. Soc*. **2014**, *35*, 3213-3218.

14 S.K. Gupta and J.Choudhury, *ChemCatChem.* **2017**, *9*, 1979-1984.

15 S. Fan, W. Dong, X. Huang, H. Gao, J. Wang, Z. Jin, J. Tang, and G. Wang, *ACS Catal.* **2017**, *7*, 243-249

16 J.B. Xia, K.W. Cormier, C. Chen, *Chem. Sci.* **2012**, *3*, 2240-2245.
